# Trends in HIV infection surveillance data among men who have sex with men in Norway, 1995-2011

**DOI:** 10.1186/1471-2458-13-144

**Published:** 2013-02-17

**Authors:** Irena Jakopanec, Andrej M Grjibovski, Øivind Nilsen, Hans Blystad, Preben Aavitsland

**Affiliations:** 1 Norwegian Institute of Public Health, Oslo, Norway; 2 Det Norske Veritas, Oslo, Norway; 3 International School of Public Health, Northern State Medical University, Arkhangelsk, Russia; 4 Epidemi, Kristiansand, Norway

**Keywords:** HIV infection, Men who have sex with men, Epidemiology

## Abstract

**Background:**

Recent reports on the growing HIV epidemic among men who have sex with men (MSM) in the EU/EEA area were accompanied by an increase of reported HIV among MSM in Oslo, Norway in 2003. Our study with data from 1995 to 2011 has described the recent trends of HIV among MSM in Norway and their socio-demographic and epidemiological characteristics.

**Methods:**

The data were collected from the Norwegian Surveillance System for Communicable Diseases. Cases were described by age, place of infection, clinical presentation of HIV infection, STI co-infection and source partner. We used simple linear regression to estimate trends over time.

**Results:**

During the study period, 991 MSM, aged from 16 to 80 years, were newly diagnosed with HIV. No significant trends over time in overall median age (36 years) were observed. Most of the MSM (505, 51%) were infected in Oslo. In the years 1995-2002, 30 to 45 MSM were diagnosed with HIV each year, while in the years 2003-2011 this increased to between 56 and 97 cases. The proportion of MSM, presenting with either AIDS or HIV illness, decreased over time, while asymptomatic and acute HIV illness increased (p for trend=0.034 or less). STI co-infection was reported in 133 (13%) cases. An overall increase of syphilis co-infected cases was observed (p for trend <0.001). A casual partner was a source of infection in 590 cases (60%).

**Conclusions:**

Though the increases described could be attributed to earlier testing and diagnosis, no change in the median age of cases was observed. This indicates that it is likely that there has been an increase in HIV infections among MSM in Norway since 2003. The simultaneous increase in STI co-infections indicates risky sexual behaviour and a potential to spread both HIV and other sexually transmitted infections.

## Background

Increases in HIV transmission among men who have sex with men (MSM) have recently been reported from several continents [1], including Europe. Data from 13 Western European countries suggested an increase of almost 100% from 1999 to 2006 [2]. In 2008, sex among MSM was the predominant mode of transmission of HIV infection in the EU/EEA area, when persons, infected in and originating from countries with generalised HIV epidemics were excluded [3].

HIV infection has been a notifiable disease in Norway since 1986 [4]. In the 1990s, the numbers of newly reported HIV infections were relatively stable, but Norway has experienced an increase in the number of HIV infected since then. Up to the 31^st^ December 2011, heterosexual transmission was reported in 52% of all reported cases and homosexual in 32% [5]. During this time, immigration to Norway has greatly increased [6] and immigrants, infected heterosexually in their country of origin before arriving to Norway, now represent 36% of all reported cases [5]. When it comes to transmission of HIV in Norway, MSM have thus been the most vulnerable population in the past decade. The first steep increase of HIV infections among MSM in Oslo was noticed in 2003, when 70% of the MSM cases diagnosed in 2003 were infected that same year or the year before [7].

Over the past few years, we have observed that MSM with newly diagnosed gonorrhoea or syphilis in Norway are increasingly co-infected with HIV [8]. This is particularly worrisome, as sexually transmitted infections (STI) might increase both susceptibility to HIV infection and enhance HIV transmission from HIV positives [9].

In order to gain a better insight into the development of the HIV epidemic among MSM in Norway, we aimed to describe the HIV diagnosed MSM over a seventeen year period, using demographic and epidemiological data. Due to an increase in HIV diagnosed MSM in 2003 [7], we were especially interested to find the differences between those MSM infected before 2003 and those infected after 2003.

## Methods

Free HIV testing is available at various settings including general practitioners, hospitals, youth clinics and STI clinics in Norway and any HIV positive test has to be confirmed through a reference laboratory. All five reference laboratories are obliged, by law [10] to report positive results anonymously to the Norwegian Institute of Public Health (NIPH) [4] when a new HIV diagnosis in Norway is confirmed. Then, a laboratory fills out a two-part reporting form with a unique reference number. The laboratory sends one part of the form to NIPH and the other part, carrying the same reference number, to the referring clinician, who provides more detailed data, including epidemiological data of the patient to the NIPH. Clinicians are also obliged by law [10], to conduct contact tracing for HIV. NIPH contacts the clinicians who fail to submit their part of the form within a month or who send an incomplete report [4].

This study is based on the Norwegian Surveillance System for Communicable Diseases database, managed at NIPH. From the database, we extracted reports on males, who reported being infected by another man (hereafter MSM cases) and were diagnosed in the years 1995 - 2011. Demographic data on each patient include gender, month and year of birth, county of residence and country of birth. A variety of epidemiological data are collected in addition, such as: presumed transmission route, time and place of infection, indications for HIV test, date of a previous (most recent) negative HIV test, clinical presentation (AIDS was defined according to the 1993 European AIDS definition in the study population, [11]), relationship to the source partner, place of infection, diagnostic site and STI infections, concurrently existing with HIV infection diagnosed (STI co-infections).

For our analyses, we grouped categories of source partner “husband or cohabitant” (58 cases) and “steady partner” (108 cases) into a common category of steady partners. Age was grouped into four categories and county of residence into four health regions. Immigrant background was assigned to first and second generation immigrants and those, adopted from abroad.

Among several potential co-existing STIs, only one is recorded at NIPH, using hierarchical criteria (1. syphilis, 2. gonorrhoea etc.). A HIV positive patient, who was also positive for Chlamydia and early syphilis (primary, secondary and early latent stage), will thus only have early syphilis recorded in addition to HIV.

A date of infection is estimated from laboratory results, previous negative tests and patients’ information. If a previous negative HIV test date is the only information available, date of infection is assigned as the midpoint of the period between that test and the first positive test [4].

Cases with “probable” year of infection had been diagnosed within three years and fulfilled the following criteria: 1. exact date of infection was known, or 2. clinical presentation as an acute HIV infection, supported by either laboratory evidence (sero-conversion) or anamnestic data, or 3. a negative test, no older than three years, was available, or 4. a clinician evaluated the anamnestic data on time of infection to be reliable. If these additional criteria were not fulfilled, but a date of infection was estimated, the cases had “uncertain” year of infection.

Among 252 cases, where the available information did not allow an estimate of the year of infection, we used clinical presentation at diagnosis, assigning 10 years to those with AIDS (median AIDS incubation time, N=53) and 3 months to those with acute HIV (N=1) [12], assigning all these to the “uncertain” year of infection group. The remaining 198 cases with asymptomatic HIV and HIV illness were excluded from estimates of time gap between year of infection and year of diagnosis.

We performed statistical analyses with Stata/SE 11.1 (Stata Corp., TX, USA). We calculated frequency distribution for descriptive analyses. We used linear regression analysis to estimate the trends over time (for STI co-infection this was only possible for syphilis). Given that the data between subsequent years may be correlated and the variability between the years may not be constant over time, the regression coefficients and their 95% confidence intervals (CI) were calculated using the Newey-West procedure.

In order to understand the differences between MSM, infected with HIV earlier in our study period compared to the period after the increase in 2003, we studied factors associated with “diagnosed in the period 2003-2011” (compared to diagnosed in the period 1995-2002) by Poisson regression with robust variance estimates. Crude and adjusted prevalence ratios (PR) with 95% CI were calculated. If there was zero variance in a category, we excluded this category from the analysis.

## Results

**Figure 1:**
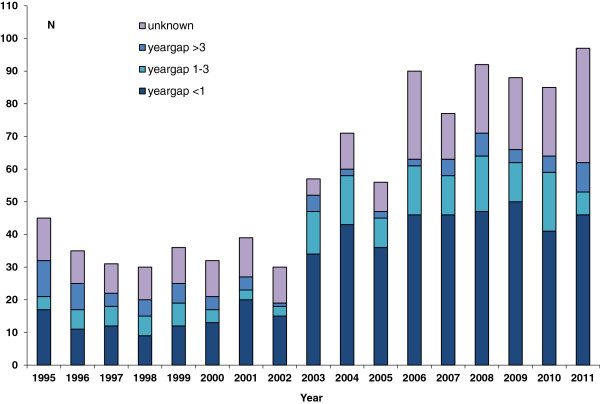
Number of MSM diagnosed with HIV infection in 1995-2011 in Norway, by year of diagnosis and time interval between this year and the estimated year of infection (“yeargap”).

In the period 1995-2011, 991 men, infected by another man, were newly diagnosed with HIV infection in Norway. While no significant trend among cases was observed until 2002, the 2003-2011 period had a significant increasing trend of HIV diagnoses among MSM (p for trend <0.001, Figure 1).

**Figure 2:**
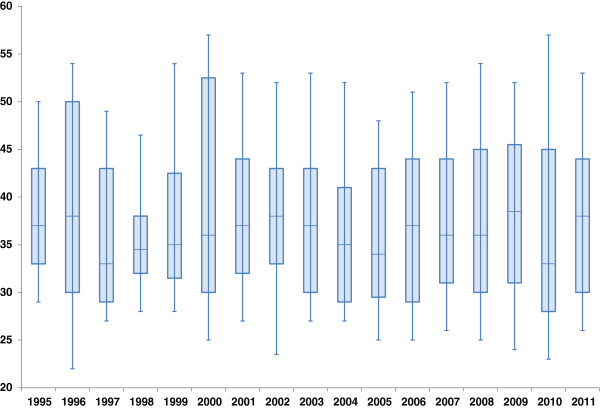
**Median age of MSM at the time of HIV diagnosis with interquartile range (boxes) and 10**^**th **^**and 90**^**th **^**age percentile in 1995-2011 in Norway, by year of diagnosis.**

The MSM were from 16 to 80 years old when diagnosed. Overall median age was 36 years (range 33-38.5 years) and interquartile range 30 - 44 years. No apparent trend in median age was observed, except for the period 1997 - 2002 (Figure 2).

Men with immigrant background represented 21% (205), and men on a temporary visit to Norway, represented 4% (37). Thus, a large majority were permanent residents of Norway, mostly residents of Oslo municipality (666, 67%), born in Norway (Table 1). The two other most frequent countries of birth were Sweden (3%) and Thailand (3%). Just above half of the cases (505, 51%) were infected in Oslo and 140 (14%) in the rest of Norway.

**Table 1:** Selected characteristics of HIV positive MSM who were diagnosed in Norway in 1995-2011, and factors associated with having been diagnosed in the years 2003-2011 compared to 1995-2002

**Demographic, epidemiological and other possibly associated factors**	**Total sample**	**Diagnosed in the years 2003-2011**	**Diagnosed in the years 1995-2002**	**Comparison of diagnosed in the years 2003-2011 vs. diagnosed in 1995-2002**		
	**N=991 (%)**	**N= 713 (%)**	**N= 278 (%)**	Crude PR* [95% CI]	Adjusted PR** [95% CI]	**p for trend**
**Age groups (years)**						
16-24	73 (7.4)	56 (7.8)	17 (6.1)	ref. group	ref. group	0.418
25-34	362 (36.5)	266 (37.3)	96 (34.5)	1.0 [0.8-1.1]	1.0 [0.9-1.1]	
35-44	323 (32.6)	221 (31.0)	102 (36.7)	0.9 [0.8-1.0]	1.0 [0.8-1.1]	
≥ 45	233 (23.5)	170 (23.8)	63 (22.7)	0.9 [0.8-1.1]	1.1 [1.0-1.3]	

**Place of birth**						
Norway	740 (74.7)	523 (73.3)	217 (78.1)	ref. group	ref. group	
Africa	23 (2.3)	18 (2.5)	5 (1.8)	1.1 [0.9-1.4]	1.3 [1.0-1.6]	
Asia	64 (6.5)	50 (7.0)	14 (5.0)	1.1 [1.0-1.3]	1.2 [1.0-1.4]	
Europe, other	92 (9.3)	71 (10.0)	21 (7.6)	1.1 [1.0-1.2]	1.1 [1.0-1.3]	
North America	13 (1.3)	11 (1.5)	2 (0.7)	1.2 [0.9-1.5]	1.2 [1.0-1.6]	NA
South/Mid America	59 (5.6)	40 (5.6)	19 (6.8)	0.9 [0.8-1.1]	1.0 [0.8-1.2]	

**Health region of residence in Norway**						
South-East***	842 (85.0)	602 (84.4)	240 (86.3)	ref. group	ref. group	NA
West	86 (8.7)	58 (8.1)	28 (10.1)	0.9 [0.8-1.1]	0.9 [0.8-1.2]	
Mid-Norway	39 (4.0)	35 (4.9)	4 (1.4)	1.2 [1.1-1.4]	1.3 [1.1-1.5]	
North	24 (2.4)	18 (2.5)	6 (2.2)	1.0 [0.8-1.3]	1.0 [0.8-1.3]	
Year gap****						
< 1 year	498 (50.2)	389 (54.6)	109 (39.2)	ref. group	ref. group	0.001
1-3 years	157 (15.8)	118 (16.5)	39 (14.0)	1.0 [0.9-1.1]	1.0 [0.8-1.1]	
>3 years	84 (8.5)	41 (5.7)	43 (15.5)	0.6 [0.5-0.8]	0.6 [0.5-0.8]	
Unknown	252 (25.4)	165 (23.1)	87 (31.3)	0.8 [0.8-0.9]	0.7 [0.6-0.9]	
**Reason for HIV test**						
Contact tracing	109 (11.0)	68 (9.5)	41 (14.8)	ref. group	ref. group	NA
Own request	328 (33.1)	261 (36.6)	67 (24.1)	1.3 [1.1-1.5]	1.3 [1.1-1.5]	
Blood donor	1 (0.1)	0	1 (0.4)	excluded	excluded	
Immigrant	11 (1.1)	7 (1.0)	4 (1.4)	1.0 [0.6-1.6]	1.0 [0.6-1.7]	
Symptoms	347 (35.0)	249 (35.0)	98 (35.3)	1.1 [1.0-1.3]	1.2 [1.0-1.4]	
Unspecified	195 (20.0)	128 (18.0)	67 (24.1)	1.1 [0.9-1.3]	1.1 [0.9-1.3]	
**Source partner**						
Steady	166 (16.7)	117 (16.4)	49 (17.6)	ref. group	ref. group	NA
Casual	590 (59.5)	442 (62.0)	148 (53.2)	1.1 [1.0-1.2]	1.1 [1.0-1.2]	
Commercial sex worker	3 (0.3)	3 (0.4)	0	excluded	excluded	
Other/unknown	232 (23.4)	151 (21.2)	81 (29.1)	0.9 [0.8-1.1]	1.0 [0.9-1.2]	
**Diagnosed by**						
GP or private specialist	412 (41.6)	315 (44.2)	97 (34.9)	ref. group	ref. group	NA
Youth/STI clinic	320 (32.3)	329 (32.1)	91 (32.7)	0.9 [0.9-1.0]	0.9 [0.9-1.0]	
Hospital/ outpatient dept.	247 (24.9)	163 (22.7)	85 (30.6)	0.9 [0.8-0.9]	0.9 [0.8-1.0]	
Other	12 (1.2)	7 (1.0)	5 (1.8)	0.8 [0.5-1.2]	0.8 [0.5-1.3]	

**Previous negative test**						
No previous test	336 (33.9)	221 (31.0)	115 (41.4)	ref. group	ref. group	NA
Tested before	588 (59.3)	425 (59.6)	163 (58.6)	1.1 [1.0-1.2]	0.9 [0.9-1.0]	
Unknown	67 (6.8)	67 (9.40)	0	excluded	excluded	
**STI co-infection**						
None reported	858 (86.6)	617 (86.5)	241 (86.7)	ref. group	ref. group	NA
Any	133 (13.4)	96 (13.5)	37 (13.3)	1.0 [0.9-1.1]	1.1 [0.9-1.2]	

* PR - prevalence ratio.

** adjusted for all the factors, listed in the Table.

**includes Oslo.

***time between infection and diagnosis in years.

For those who were infected abroad (248, 25%), predominant countries of HIV acquisition were Thailand (34 cases), Spain (33), USA (26 cases) and Germany (23 cases). Some (109, 11%) could not identify where the infection was acquired. Among those infected abroad, 39 had tested positive prior to arrival in Norway. Among 251 MSM, born abroad, 105 acquired HIV infection abroad, compared to 143 out of 740 MSM, born in Norway. Their median age at diagnosis was lower than for Norwegian born men (31 years vs. 38 years).

**Figure 3:**
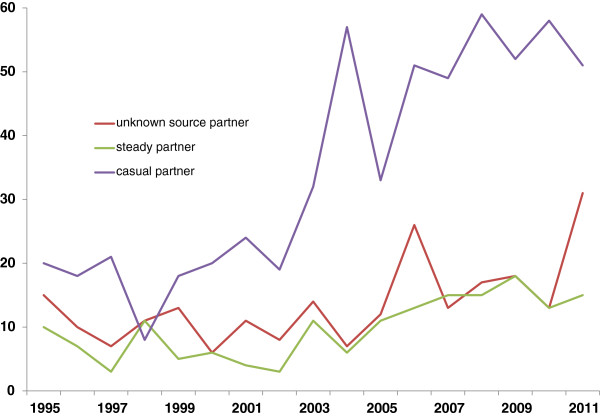
Number of MSM diagnosed with HIV infection in 1995-2011 in Norway, by year of diagnosis and assumed source partner.

**Figure 4:**
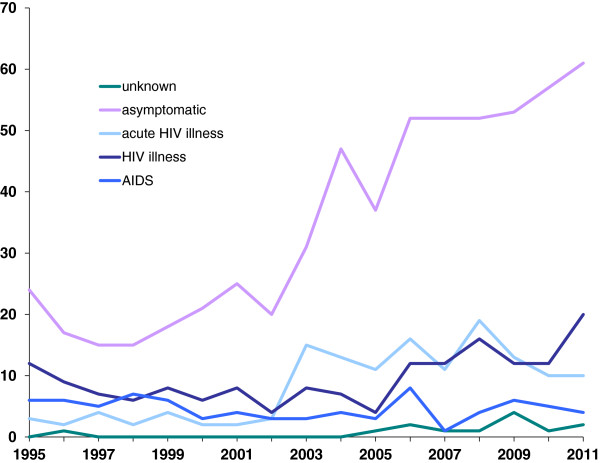
Number of MSM diagnosed with HIV infection in 1995-2011 in Norway, by year of diagnosis and clinical symptoms.

Half of the cases were infected less than a year prior to being diagnosed (Table 1, Figure 1). The increase in infections in 2003 and 2004 (Figure 1) coincides with an increase of cases, infected by a casual partner (p for trend <0.001) (Figure 3). The majority of cases were asymptomatic at the time of diagnosis (597, 60%, Figure 4). Furthermore, the proportion of MSM presenting with either AIDS or HIV illness, decreased over time, however, the proportion of both asymptomatic and those with acute HIV illness increased (all with p for trend=0.034 or less).

Of the 133 (13%) cases with concurrent HIV and STI infections, syphilis was reported in 50 (3%). Among the remaining 83, 29 had gonorrhoea. Of those 54, who had neither syphilis or gonorrhoea, 21 had hepatitis B, 16 had Chlamydia, 6 had genital warts, 5 had genital herpes and 6 had ”other, unspecified STI”.

From 1995 to 1999, one HIV and syphilis co-infection among MSM was reported (in 1998). In 2000, a year after an outbreak of syphilis among MSM in Norway [8], 5 cases (15.6% of all HIV MSM cases that year) with co-infection were reported. From 2001, the numbers of HIV and syphilis co-infected MSM vary (0-9 cases, 0-9% of all HIV cases), but there is an overall increase of syphilis co-infected cases (p for trend <0.001). The number of MSM with any other STI co-infection (excluding syphilis), also increased in time (p for trend = 0.016, lowest 2 in 1998, highest 13 in 2010).

In the crude analysis, MSM diagnosed in the years 2003-2011 were more likely to live in Mid-Norway, were tested at their own request (compared to contact tracing) when found positive, and were more likely to have been tested before compared to those, diagnosed in 1995-2002. MSM diagnosed in 2003-2011 were also less likely to have been diagnosed more than 3 years since infection, to have an unknown year of infection and to be tested at a hospital or outpatient clinic (compared to a general practitioner’s office). After adjustment for other factors (all listed in the Table 1), findings were similar, and in addition, MSM diagnosed in 2003-2011 seemed to be more likely born abroad compared to those, diagnosed in 1995-2002. The results were significant for MSM from Asia and suggested similar effect for MSM from other countries of Europe (p=0.051), Africa (p=0.055) and North America (p=0.090).

**Figure 5:**
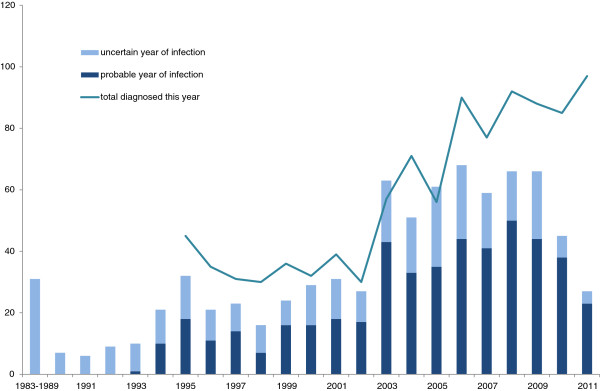
**Number of MSM diagnosed with HIV infection in 1995-2011 in Norway, by their estimated year of infection (bars) (N=793) and by year of diagnosis (line) (N=991).** For the incidence bars, we excluded 198 cases with unknown year of infection and grouped 31 cases with uncertain year of infection before 1990 in one bar. Note that existing bars will increase in the coming years as new cases infected in those years are diagnosed, especially in the most recent years. The incidence bars for the years before 1995 do not include those, diagnosed before 1995.

A “probable” year of infection was available for 479 (48 % of total sample) cases and an “uncertain” year of infection for a further 314 (32 %) cases (Figure 5). The number of HIV infections increased markedly in 2003 and remained higher afterwards. The mean time between diagnosis and infection for these 793 (80%) of cases was 7.1 months (interquartile range 2.7 - 23.2). In 2003, the number of cases infected more than doubled compared to a year before (from 27 to 63) and remained at high levels afterwards.

## Discussion

We have observed an increase in number of HIV diagnoses among MSM in Norway in recent years, particularly after 2002. The majority of MSM were infected in Oslo and they were of Norwegian origin. Although HIV is diagnosed mainly in asymptomatic MSM, our results suggest that most of the cases diagnosed after 2002, have been infected less than a year before being diagnosed. We observed a simultaneous increase of STI co-infections, which is worrisome, as there is a potential for spread of both HIV and other STI.

The possibility for NIPH to send a reminder to the diagnosing clinicians, if they fail to report a newly identified HIV infection, contributes to a high completeness of information [4]. There were no changes in the reporting system in our study period and reporting delays are rare in Norway [4]. Due to anonymous reporting, cases could be reported twice if they are re-tested; however, laboratories do check for duplicates in the same region before submitting a report and further efforts to detect and remove duplicates are done at NIPH [4]. It is worth emphasizing that all risk behaviour data, obtained by clinicians from their patients, such as, for example, presumed transmission route, HIV infection source partner and other risk factors for HIV transmission (for example, injecting drug use), may be subject to recall and reporting biases. Since some MSM might not be open with their doctor about the way they were infected, we may have missed some cases which have been classified in other transmission groups. As the system records any infection not reported previously, the HIV positive person who immigrated to Norway is also included, if they were tested in Norway (44 in our study population). Merging STI into a common outcome has its limitations, as some STIs might be recent (acute) and others, such as hepatitis B, could be chronic with different risk factors.

The HIV surveillance data monitor the incidence of new *diagnoses* rather than new *infections* and will be thus largely influenced by HIV testing uptake. Since specific tests to distinguish between the early and late infection [13] are not yet routinely applied in HIV diagnostics and reporting in Norway, it is difficult to distinguish between the current increase in HIV transmission and a backlog of cases, undiscovered in the previous years.

While no systematically collected data on testing activity among MSM in Norway in our study period exist, some data suggest satisfactory testing rates. In a 2003 study where MSM were approached at several gay venues in Oslo, 87% have answered they had ever been tested for HIV (76% in 1990, 80% in 1998) [14]. In a 2007 survey in Norwegian language that recruited respondents among visitors to a gay Internet site [15], 69% of 2598 responding MSM reported that they had been tested for HIV at least once and the proportion who tested positive was 4% (data not published). In a more recent internet based cross-European MSM survey from 2010, 67% of responding MSM in Norway reported having received the HIV test result at least once, of which 3.5% were positive [16]. A third has received the result of their HIV test in the last 12 months in 2010 survey [17] and 37% in 2007 survey (data not published). We can conclude testing rates among MSM in Norway are satisfactory, though we should be aware the test uptake is likely lower among those who are infected [18].

Though information on time of infection is subject to bias, the assumption that there is an actual increase in recently infected cases is supported by the median age showing no significant increase over time [19]. Furthermore, results from the internet based behavioural survey in 2010 have revealed worrisome risky behaviour, as 54% of MSM respondents who had engaged in sex with a non-steady partner had had anal intercourse without a condom in the past year and more than 30% said they did not use a condom the last time they had anal sex with a casual partner [16].

In order to clarify the described increasing trends, our findings call for improvements in HIV monitoring in Norway. Regular monitoring of HIV prevalence using anonymous unlinked studies, as well as monitoring testing trends and MSM behaviour in Norway are recommended. Introduction of CD4 count in surveillance data and approaches to reduce potential double-reporting (such as Soundex code) should be considered, as more and more MSM live with HIV in Norway and the risk of double reporting after re-testing is increasing.

We aimed to estimate year of infection from the information available, though important limitations (see above) with this approach exist, since for 20% of diagnosed cases the year of infection could not be estimated. Thus, our time intervals between infection and first diagnosis are underestimated as those excluded 20% are likely to have an interval longer than 7 months. A French study based on surveillance data from 2004-2007 reported much longer time (median 25 months, interquartile range 5-55) [12]. Had we assigned an overestimate of 10 years to the remaining 20% of cases, our median would increase to 12 months, indicating MSM in Norway are on average being diagnosed earlier than French.

The surveillance systems of new HIV diagnoses in European countries have varying characteristics, which makes it difficult to compare our results, however; similar increasing trends in diagnoses of HIV infections among MSM of all age groups have recently been reported in Belgium (1999-2008) [20], Germany (1999-2005) [21] and the UK outside of London (1997-2004) [22]. Contrary to this, data from London and the Amsterdam STI clinic (1991-2004) suggested that an increase in HIV incidence was observed specifically among MSM aged 35 years and older [22,23]. Median age in our study seemed to be similar to the one in Belgium and Denmark (1990-2005) [24] with median age 37.

Similar to our findings, in Belgium, 11% of HIV positive MSM had an STI co-infection in 2008 (data provided by participating AIDS Reference Centers) [20]. In the period 2003-2007, as many as 31% of the 1462 newly HIV diagnosed MSM in Spain (data from 19 HIV/STI clinics) were diagnosed with a concurrent STI [25]. An increase from 5% (2001) to 7% (2003) in concurrent syphilis and HIV was reported from Germany [26]. Syphilis outbreaks including HIV positive MSM were described in metropolitan areas of Western Europe (London, Paris, Dublin, Hamburg) with MSM who were mostly aware of their HIV status [27].

HIV was the most frequently reported STI among MSM in Norway in 2006 [8,28]. With rising HIV prevalence, MSM are more likely to have sex with an HIV positive man than a man with any other acute STI [29]. In addition, the prevalence of STI, which may increase the risk of HIV transmission, is now higher, and per-contact probability of HIV transmission has been evaluated as similar to that of the pre-HAART era [30]. Primary HIV infection may have a larger role in the dynamics of HIV transmission than previously assumed, due to a higher viral load and higher infectiousness [31]. Our results suggest similar trends in Norway.

HIV transmission is predominantly affecting domestic-born MSM [20,24,32], however, similar to other Western countries, immigration has more than doubled in Norway since 2000[33] and foreign born MSM are becoming more represented in the recent years. Foreign born MSM are a “difficult to reach”, heterogeneous and vulnerable population, likely diagnosed younger than domestic-born MSM [32]. According to guidelines issued by the Norwegian Directorate of Health, immigrants from high HIV prevalent areas should be offered a free confidential HIV test [34] and support and treatment, if found positive. Nonetheless, less than 1% of MSM were diagnosed due to immigrant screening in our study, which is low compared to the group of heterosexuals, infected before arrival to Norway, who represent more than a third of all newly diagnosed HIV cases in Norway [16]. General practitioners should be aware that some men might be reluctant to provide details of their sexual behaviour to their clinician, particularly if they originate from cultures, where such behaviour is not acceptable. Specific preventive and testing measures, targeted at foreign born MSM, should be evaluated.

Those diagnosed in the years 2003-2011, were more likely to be tested at their own request compared to contact tracing and less likely to be diagnosed at hospital or outpatient clinic than by their general practitioner. With a simultaneous decrease of AIDS and HIV illness, this might have been the effect of a 2005 recommendation to general practitioners for annual HIV and STI testing of MSM in Norway [35] and other MSM targeted public health campaigns, encouraging HIV testing. Thus, risky behaviour among MSM, might have been accompanied by improved awareness on importance of testing and early capture of infections. We concur with the recommendations from 2005 and emphasize the need to target MSM with immigrant background, as well as to conduct thorough partner notification in the general practitioner’s setting.

Similar increasing HIV trends are described among MSM around Europe and a coordinated approach in HIV monitoring, prevention and control, should be complemented with identification of new prevention strategies. In the light of failing preventive measures (such as promotion of safe sex practices and condom use), pre-exposure prophylaxis is a promising biomedical intervention, however still controversial due to possible side effects on healthy men, poor adherence among HIV negative, costs, change in sexual behaviour and resistance issues to name a few [36,37]. Further studies are on the way to evaluate the effectiveness of this approach [37]. Effectiveness of male circumcision on HIV transmission among MSM remains unknown [38].

Reasons why those living in Mid-Norway are more frequent among cases from 2003-2011 are not clear. Recently, rapid testing has become available countrywide, in both health centers and specialized clinics, as well as in outreach centers. In the latter, this is an assumed low threshold offer that might increase testing uptake among high risk MSM. It remains unanswered if current healthcare services are sufficiently targeting foreign born MSM with testing and information on STI prevention, though this low threshold offer could make a difference.

## Conclusions

Our data suggest continuing spread of HIV among MSM in Norway. On average, MSM are being diagnosed early after infection, however an increasing proportion are co-infected with an STI. This calls for prompt and effective preventive measures as many past preventive efforts seem to have been insufficient or failing. At the same time, current monitoring of HIV infections among MSM has many limitations. In the light of the increase in HIV diagnoses among MSM, this calls for urgent improvements and more research on HIV prevalence, regular testing activity and risky behaviour in this population.

## Competing interests

The authors declare no competing interests. The entire funding was provided by the Norwegian Institute of Public Health. No commercial funding was received to conduct this study.

## Authors' contributions

IJ drafted the manuscript. ØN collected and entered data and contributed with interpretation. IJ, AG, PA and HB contributed to the design of the study, analysis and interpretation. All authors critically reviewed and approved the final version of this paper for publication.

## Pre-publication history

The pre-publication history for this paper can be accessed here:

http://www.biomedcentral.com/1471-2458/13/144/prepub
